# Characterization of PSA dynamics and oncological outcomes in patients with metastatic hormone-sensitive prostate cancer treated with androgen receptor signaling inhibitors

**DOI:** 10.1007/s10147-024-02676-z

**Published:** 2024-12-10

**Authors:** Yasutaka Yamada, Kodai Sato, Shinichi Sakamoto, Takuya Tsujino, Sinpei Saito, Kazuki Nishimura, Tatsuo Fukushima, Ko Nakamura, Yuki Yoshikawa, Tomohisa Matsunaga, Ryoichi Maenosono, Manato Kanesaka, Takayuki Arai, Tomokazu Sazuka, Yusuke Imamura, Kazumasa Komura, Kazuo Mikami, Kazuyoshi Nakamura, Satoshi Fukasawa, Kazuto Chiba, Yukio Naya, Maki Nagata, Atsushi Komaru, Hiroomi Nakatsu, Haruhito Azuma, Tomohiko Ichikawa

**Affiliations:** 1 Department of Urology, Chiba University Graduate School of Medicine, 1-8-1 Inohana, Chuo-ku, Chiba-City, Chiba 2608670 Japan; 2 Department of Urology, Osaka Medical and Pharmaceutical University, Osaka, Japan; 3 Department of Urology, Chibaken Saiseikai Narashino Hospital, Chiba, Japan; 4 Department of Urology, Kimitsu Chuo Hospital, Chiba, Japan; 5 Department of Urology, Funabashi Municipal Medical Center, Chiba, Japan; 6 Department of Urology, Fukaya Red Cross Hospital, Saitama, Japan; 7 Department of Urology, Teikyo University Chiba Medical Center, Chiba, Japan; 8 Department of Urology, Yokohama Rosai Hospital, Kanagawa, Japan; 9 Prostate Center and Division of Urology, Chiba Cancer Center, Chiba, Japan; 10 Department of Urology, Asahi General Hospital, Chiba, Japan

**Keywords:** Metastatic hormone-sensitive prostate cancer, Androgen receptor signaling inhibitor, Bicalutamide, PSA nadir, Time to PSA nadir, PSA dynamics

## Abstract

**Background:**

This study investigated the characteristics of prostate-specific antigen (PSA) dynamics when androgen receptor signaling inhibitor (ARSI), or vintage agent (bicalutamide) was used for patients with metastatic hormone-sensitive prostate cancer (mHSPC).

**Patients and methods:**

A total of 213 mHSPC patients from each of the ARSI and bicalutamide groups treated between 2015 and 2022 were selected from multiple institutions using propensity score-matched analysis to align backgrounds. PSA progression-free survival (PFS) and overall survival (OS) were assessed. PSA level at 3 months, PSA nadir level, and time to PSA nadir were examined to analyze of PSA kinetics.

**Results:**

ARSI treatment significantly improved PSA PFS compared to bicalutamide (*P* = 0.0063), although no significant difference in OS was seen (*P* = 0.3134). No significant differences were observed between treatment groups in median PSA levels at 3 months (1.47 vs 0.52 ng/ml, *P* = 0.3042) or PSA nadir levels (0.263 vs 0.1345 ng/ml, *P* = 0.1228). Bicalutamide treatment demonstrated longer time to nadir than ARSI in progression-free cases (median: 243 vs 213.5 days, *P* = 0.0003). Survival tree analysis found that PSA nadir ≤ 1.5 ng/ml and time to nadir ≥ 145 days were the optimal cut-offs for best stratifying OS with bicalutamide, while PSA nadir ≤ 0.45 ng/ml and time to nadir ≥ 70 days were optimal with ARSI.

**Conclusion:**

No significant differences in PSA response was seen between groups; however, distinct optimal cut-offs were demonstrated for PSA nadir and time to nadir. The present findings will be useful for optimal PSA monitoring for mHSPC patients and for early identification of poor-prognosis populations.

**Supplementary Information:**

The online version contains supplementary material available at 10.1007/s10147-024-02676-z.

## Introduction

Prostate cancer (PCa) is the most frequently diagnosed male cancer and the second leading cause of death in United States after lung cancer [1]. Although the treatment of metastatic PCa is evolving rapidly with the emergence of novel therapeutic approaches such as prostate-specific membrane antigen (PSMA)-targeted radiopharmaceuticals and inhibitors of poly(ADP-ribose) polymerase (PARP), the clinical outcomes for this disease remain unfavorable [2]. Androgen receptor signaling inhibitors (ARSI) (e.g., apalutamide, enzalutamide, darolutamide, abiraterone acetate) have recently become a mainstay of treatment option for advanced PCa including metastatic hormone-sensitive prostate cancer (mHSPC), non-metastatic castration-resistant prostate cancer (nmCRPC), and metastatic CRPC (mCRPC) [3–6]. Additional effects of ARSI to androgen-deprivation therapy (ADT) have been examined and risk reduction of death was shown as an initial treatment for patients with mHSPC [3, 7–9]. The National Comprehensive Cancer Network (NCCN) guideline for prostate cancer classifies mHSPC disease into high or low volume and synchronous or metachronous metastases and recommends treatment options for each disease status [10]. ARSI treatment is a standard therapeutic agent in all disease states, although the option of combining with radiotherapy or docetaxel does exist. Conventional vintage therapies (e.g., bicalutamide, flutamide, nilutamide) are, thus, becoming obsolete in advanced PCa.

Given that the majority of patients with mHSPC may progress to mCRPC disease [11], detecting disease progression sensitively is crucial when providing treatment with ARSI agents. Prostate-specific antigen (PSA) kinetics have previously been validated as a promising prognostic marker and have demonstrated a robust role in predicting survival during hormone therapy [12–14]. PSA response at early timing and achievement of lower PSA nadir value have been highlighted as a useful surrogate prognostic marker and found to be correlated with superior survival from a sub-analysis of a clinical trial [15]. PSA response after initiation of ARSI administration can, therefore, provide an easily available and rapid prognostic marker to predict subsequent outcomes. However, little evidence has been accumulated on the characteristics of PSA dynamics in ARSI treatment as compared to vintage treatment and the prognostic impact of the PSA response.

The present study explored PSA dynamics when treated with ARSI or vintage treatment for patients with mHSPC and examined their differences. Furthermore, optimal threshold of PSA nadir and time to nadir period to predict survival were validated in each treatment groups. Our findings will ultimately help build a better understanding of PSA monitoring and detect unfavorable patients’ population at early timing in the ARSI era.

## Patients and methods

### Patients

Patients with newly diagnosed mHSPC treated between 2015 and 2022 at multiple institutions were retrospectively included in our study. All patients received prostate biopsy examination and were diagnosed with adenocarcinoma of the prostate. Metastatic sites were identified using whole-body computed tomography (CT) and technetium-99 m-methylene diphosphate (^99m^Tc-MDP) bone scintigraphy. All patients received ADT with surgical or pharmacological castration in combination with a vintage nonsteroidal antiandrogen agent (bicalutamide) or a novel hormonal therapy (enzalutamide, apalutamide, or abiraterone acetate) as first-line treatment. No patients underwent upfront chemotherapy.

To adjust for differences in patient characteristics between the ARSI and vintage treatment groups, propensity score matching (PSM) was employed using major clinical parameters (age, initial PSA (iPSA), International Society of Urological Pathology grade group (ISUP GG), and visceral metastasis status). After PSM, a total of 426 patients (213 patients from each group) were selected for the analysis in this study. The present study was approved by the institutional review boards (approval no. M10238 and RIN750-2571).

### Clinical parameters

The following clinical records were collected for this study: age at diagnosis, iPSA value, ISUP GG, TNM classification, extent of disease (EOD) score, location of visceral metastasis, and baseline peripheral blood examinations (hemoglobin (Hb), lactate dehydrogenase (LDH), alkaline phosphatase (ALP), and albumin (Alb)). High-/low-volume and high-/low-risk disease were determined according to the criteria in the CHAARTED [16] and the LATITUDE [9] trials, respectively.

### Oncological outcomes

The primary outcomes were PSA progression-free survival (PSA PFS) and overall survival (OS). Prostate Cancer Working Group (PCWG) 2 or 3 criteria [17, 18] were used to determine the PSA PFS. In terms of PSA kinetics, PSA level and reduction rate at 3 months after treatment initiation, PSA nadir level, and time to PSA nadir were examined in this study.

### Statistical analysis

Student’s *t* test and the *χ*^2^ test were used for head-to-head comparisons across the two groups. The Kaplan–Meier method and Cox proportional hazards model were utilized to determine the statistical significance of outcomes and prognostic factors. PSM was employed to match clinical factors in the patient background. JMP16 software (SAS Institute, Cary, NC, USA) was used to conduct statistical analyses in this study. Statistical significance was determined at *P* < 0.05 in this study.

### Determination of the optimal threshold for PSA nadir value and time to nadir

The optimal PSA nadir and time to nadir threshold that best stratified OS in patients treated with vintage drugs and ARSI were calculated using a survival tree analysis. As a type of decision tree used for analyzing survival data, the survival tree divides patient data into subgroups based on the relationship between risk factors and survival time, analyzing the survival probabilities within each subgroup. In this study, hyperparameter tuning was performed to split the data into two subgroups. This approach maximizes the difference in survival probabilities between the two groups. The value of the risk factor that maximizes the difference in survival times between the two groups is identified as the threshold. Using this method, the optimal PSA nadir and time to nadir thresholds were determined.

## Results

### Patients background in ARSI or vintage treatment groups

From the original cohort including 304 patients for ARSI and 295 patients for vintage treatment (Table S1), we selected 426 mHSPC patients (213 patients from each group) using PSM to align their background. Characteristics of patients are summarized in Table 1. Median age and iPSA level were 74 years old and 209.5 ng/ml in the ARSI group, and 75 years old and 169.98 ng/ml in the vintage group, respectively. The percentage of ISUP GG5 was 66.2% in the ARSI group and 67.1% in the vintage group. Visceral metastasis was observed in 19.3% of the ARSI group and 16.5% of the vintage group. High-volume and high-risk disease were seen in 63.4% and 69% of the ARSI group and 62% and 65.3% of the vintage group. No significant differences were observed between the ARSI and vintage groups except for cN stage (*P* = 0.0048) and serum ALP level (*P* = 0.0375). In the ARSI group, abiraterone acetate was the most commonly used upfront treatment (55.4%) followed by apalutamide (28.2%) and enzalutamide (16.4%). In the vintage group, all patients in this study received bicalutamide administration. Median follow-up period was 24 months, and 93 patients died during this study.

**Table 1:** Characteristics of patients after Propensity Score Matching

	Treatment groups		*P* value*
	ARSI (*n* = 213)	Vintage (*n* = 213)	
Median age (range), years	74 (52–91)	75 (51–93)	0.7952
Median initial PSA (range), ng/ml	209.5 (4.9–17,300)	169.98 (1.15–19,908)	0.6741
ISUP GG, *n* (%) ≤ 3	8 (3.8)	6 (2.8)	0.8371
4	64 (30)	64 (30)	
5	141 (66.2)	143 (67.1)	
T stage, *n* (%) ≤ 3b	141 (66.2)	138 (64.8)	0.7598
≥ 4	72 (33.8)	75 (35.2)	
N stage, *n* (%)			0.0048
positive	159 (74.6)	132 (61.9)	
M stage, *n* (%)			0.4475
1a/1b/1c	15 (7)/157 (73.7)/41 (19.3)	15 (7)/163 (76.5)/35 (16.5)	
EOD score ≥ 2	101 (47.4)	94 (44.1)	0.496
Location of visceral metastasis, *n* (%)			
Liver	6 (2.8)	5 (2.3)	0.7598
Lung	37 (17.4)	37 (17.4)	1
Both	5 (2.3)	2 (0.9)	0.2453
Baseline peripheral blood markers, median			
Hb (range), g/dL	13.3 (5.5–17.7)	13.5 (5.5–18.8)	0.1961
LDH (range), U/L	194 (128–2189)	192 (125–1715)	0.3174
ALP (range), U/L	261 (52–9813)	451.7 (86–14,290.88)	0.0375
Alb (range), g/dL	4 (2.5–4.9)	4.1 (2.1–5.1)	0.195
High volume, *n* (%)	135 (63.4)	132 (62)	0.7638
High risk, *n* (%)	147 (69)	139 (65.3)	0.4092
initial treatment, *n* (%)			-
Apalutamide	60 (28.2)	0 (0)	
Enzalutamide	35 (16.4)	0 (0)	
Abiraterone acetate	118 (55.4)	0 (0)	
Bicalutamide	0 (0)	213 (100)	
PSA progression, *n* (%)	54 (25.4)	108 (50.7)	-
Death, *n* (%)	32 (15)	61 (28.6)	-

*PSA* prostate-specific antigen, *ISUP GG* International Society of Urological Pathology grade group, *Hb* hemoglobin, *LDH* lactate dehydrogenase, *ALP* alkaline phosphatase, *Alb* albumin, *ARSI* androgen receptor signaling inhibitor

### PSA kinetics in the ARSI and vintage treatment group

**Fig. 1:**
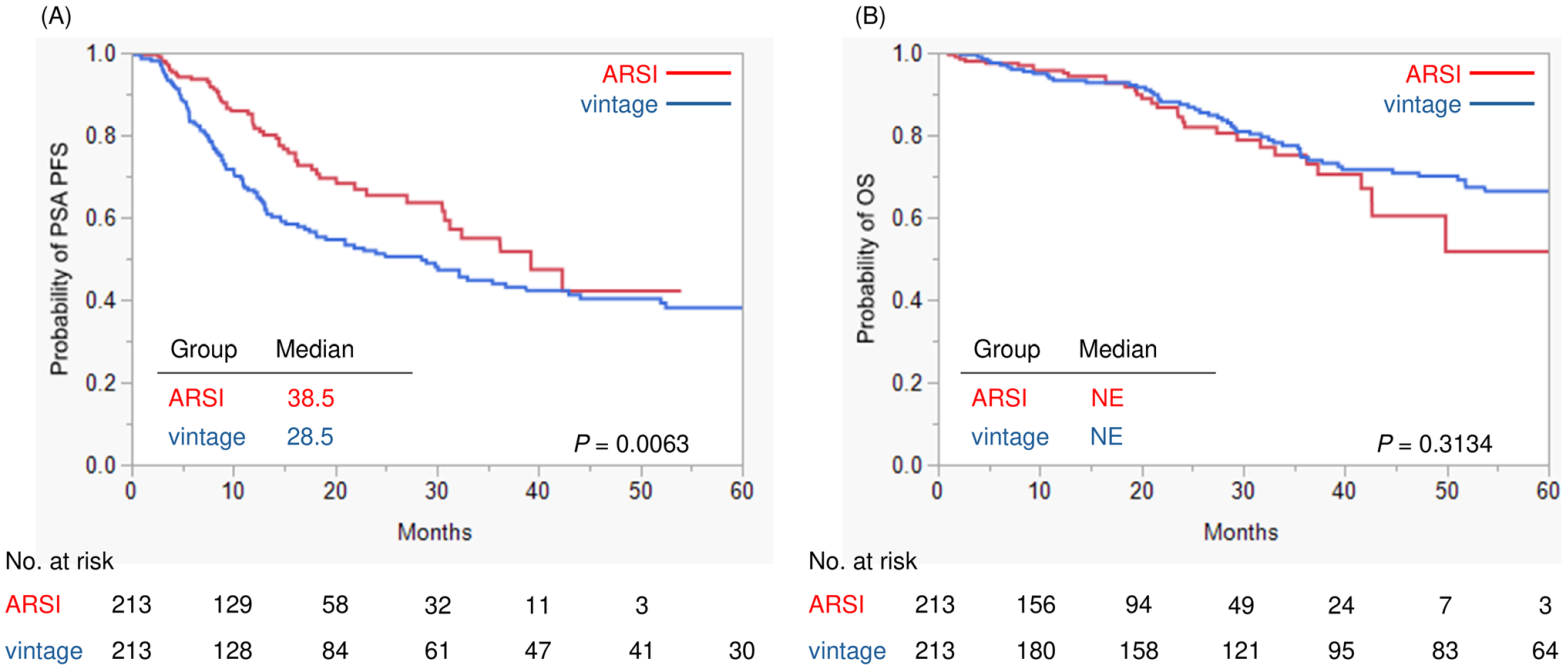
Comparison of survival between ARSI and Vintage treatment. **A** PSA PFS, **B** OS

Kaplan–Meier analysis showed that patients who received an ARSI as the initial treatment was associated with prolonged PSA PFS as compared to those who received a vintage agent (*P* = 0.0063) (Fig. 1A). Median PSA PFS was 38.5 months in the ARSI group and 28.5 months in the vintage group, respectively. Regarding OS, no significant difference was seen between two groups (*P* = 0.3134) (Fig. 1B).

In terms of PSA response in the early stage of treatment, medial PSA levels at 3 months were 0.52 ng/ml in the ARSI group and 1.47 ng/ml in the vintage groups (*P* = 0.3042). On the other hand, ARSI treatment group achieved a lower PSA level at 3 months than vintage treatment group when the cut-off PSA value was set at ≥ 0.5 ng/ml (Table 2). A PSA level < 0.5 ng/ml at 3 months was seen in 49.1% of patients in the ARSI group and 36.9% of patients in the vintage group (*P* = 0.0205) at 3 months. When looking at the deeper PSA response (PSA nadir < 0.2 ng/ml), no significant differences were found between two groups.

**Table 2:** PSA dynamics in each treatment group

	Treatment groups		*P* value*
	ARSI (*n* = 213)	Vintage (*n* = 213)	
PSA level at 3 months (ng/ml)			
median (range)	0.52 (0.008–1381)	1.47 (0.008–1190)	0.3042
< 0.02 ng/ml	9/171 (5.3%)	10/179 (5.6%)	0.9161
< 0.1 ng/ml	38/171 (22.2%)	35/179 (19.6%)	0.539
< 0.2 ng/ml	50/171 (29.2%)	44/179 (24.6%)	0.3256
< 0.5 ng/ml	84/171 (49.1%)	66/179 (36.9%)	0.0205
< 1 ng/ml	102/171 (59.6%)	79/179 (44.1%)	0.0036
< 2 ng/ml	122/171 (71.3%)	98/179 (54.7%)	0.0001
≥ 2 ng/ml	49/171 (28.7%)	81/179 (45.3%)	0.0001
PSA reduction at 3 months (%)			
median (range)	99.58 (−255–99.99)	99.35 (−332.9–99.99)	0.5539
≥ 50%	166/171 (97.1%)	169/179 (94.4%)	0.1552
≥ 70%	160/171 (93.6%)	165/179 (92.2%)	0.4582
≥ 90%	148/171 (86.5%)	158/179 (88.3%)	0.8726
≥ 99%	102/171 (59.6%)	96/179 (53.6%)	0.256
nadir PSA level (ng/ml)			
median (range)	0.1345 (0–4343.35)	0.263 (0–534.6)	0.1228
< 0.02 ng/ml	58/197 (29.4%)	50/193 (25.9%)	0.9053
< 0.1 ng/ml	89/197 (45.2%)	79/193 (40.9%)	0.2305
< 0.2 ng/ml	104,197 (52.8%)	90/193 (46.6%)	0.1052
< 0.5 ng/ml	125/197 (63.5%)	110/193 (56.9%)	0.1925
< 1 ng/ml	136/197 (69%)	128/193 (66.3%)	0.5666
< 2 ng/ml	147/197 (74.6%)	145/193 (75.1%)	0.9075
≥ 2 ng/ml	50/197 (25.4%)	48/193 (24.9%)	0.9075
time to nadir (days), median (range)			
all	200 (0–1620)	203 (0–2298)	0.024
progression cases	185.5 (0–624)	175.5 (0–987)	0.7509
progression-free cases	213.5 (7–1620)	243 (0–2298)	0.0003

*PSA* prostate-specific antigen, *ARSI* androgen receptor signaling inhibitor

In terms of PSA reduction rate at 3 months, no significant difference was observed between the two groups (Table 2). PSA reduction ≥ 90% was seen in 86.5% of the ARSI group and 88.3% of the vintage group (*P* = 0.8726).

Furthermore, no significant difference was observed in nadir PSA level with median PSA nadir of 0.1345 ng/ml in the ARSI group and 0.263 ng/ml in the vintage group (*P* = 0.1228) (Table 2). No significant differences were seen with all cut-off values of PSA nadir (Table 2). A nadir PSA level < 0.2 ng/ml was seen in 52.8% of the ARSI group and 46.6% of the vintage group (*P* = 0.1052).

Time to PSA nadir was shorter in the ARSI treatment group (213.5 days) than in the vintage group (243 days; *P* = 0.0003) among progression-free cases, although no significant difference was observed in patients with progression (185.5 days vs 175.5 days, respectively; *P* = 0.7509).

Additional analysis using iPSA values (> 200 ng/ml or ≤ 200 ng/ml) was performed. The median PSA values at 3 months were 0.708 ng/ml for the ARSI and 4.413 ng/ml for the vintage in patients with iPSA > 200 ng/ml, whereas they were 0.4365 ng/ml for the ARSI and 0.448 ng/ml for the vintage in those with iPSA ≤ 200 ng/ml (Table S2 and S3). With respect to nadir PSA level, the rate of achieving PSA < 0.2 ng/ml was higher in the ARSI group (49.5%) than in the vintage group (32.2%) in patients with iPSA > 200 ng/ml (*P* = 0.0151) although no significant difference was seen in those with iPSA ≤ 200 ng/ml, indicating the ARSI treatment is more likely to have an evident PSA-suppressing effect when iPSA levels are high (Table S2 and S3).

### Cut-off values for nadir PSA level and time to nadir to predict OS

**Fig. 2:**
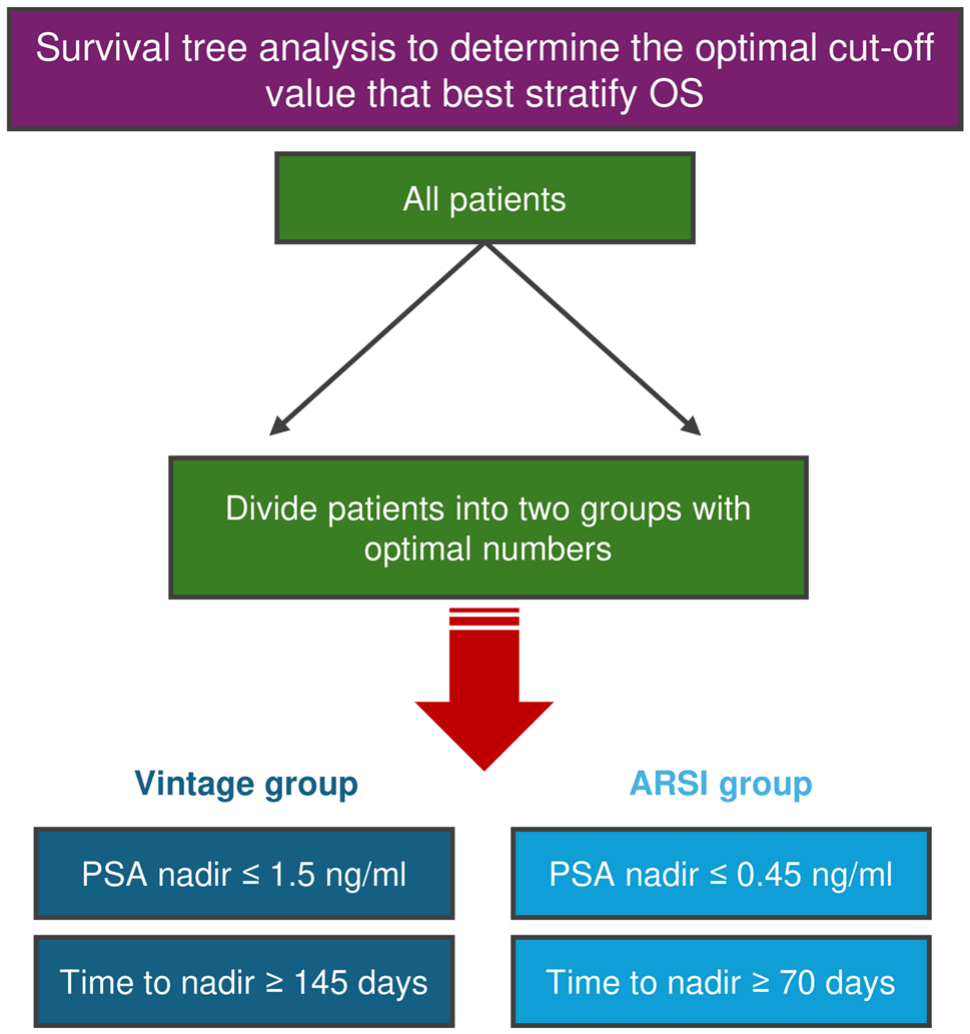
Methodology of survival tree analysis

We investigated the cut-off values for nadir PSA and time to nadir to best stratify OS in each treatment group using survival tree analysis (Fig. 2). As a result, the optimal cut-off nadir PSA level was 1.5 ng/ml for the vintage group and 0.45 ng/ml for the ARSI group (Fig. 2A). The optimal cut-off time to nadir was 70 days for the vintage group and 145 days for the ARSI group.

**Fig. 3:**
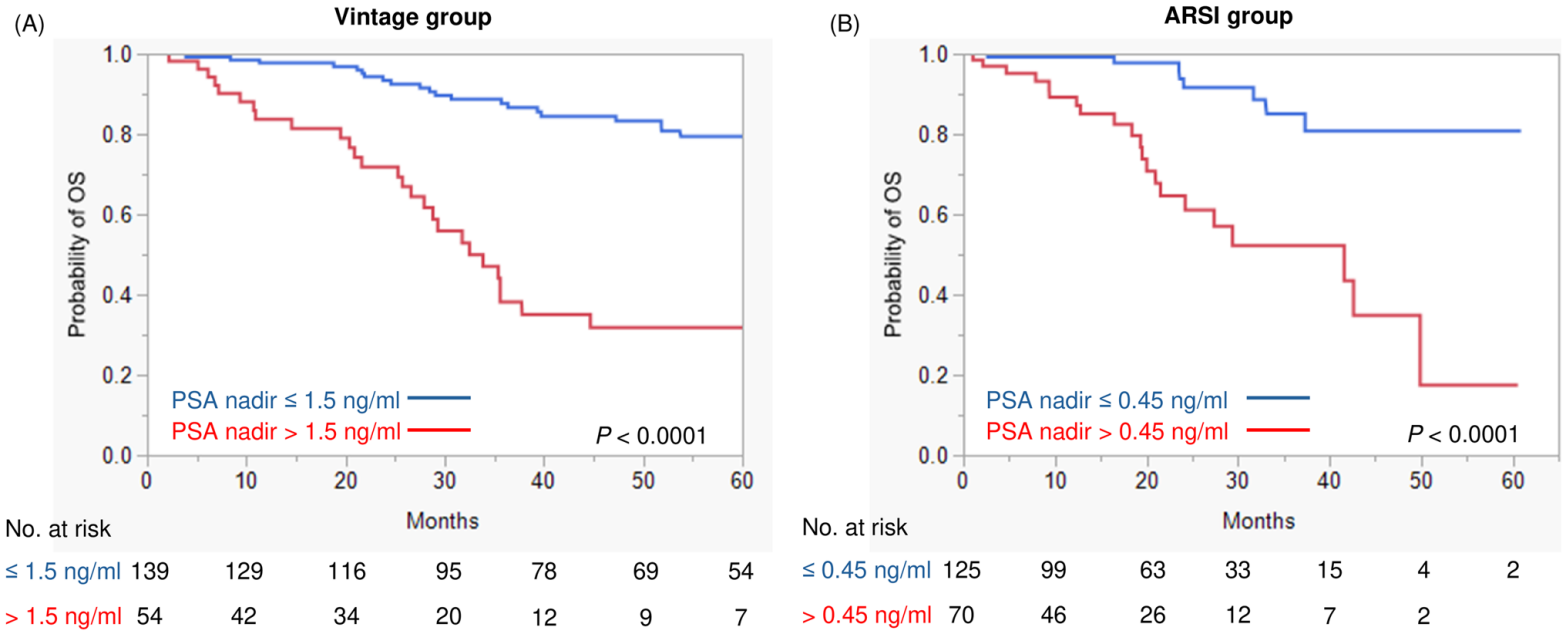
Survival analysis divided by optimal cut-off PSA nadir level. **A** Vintage treatment group. **B** ARSI treatment group

**Fig. 4:**
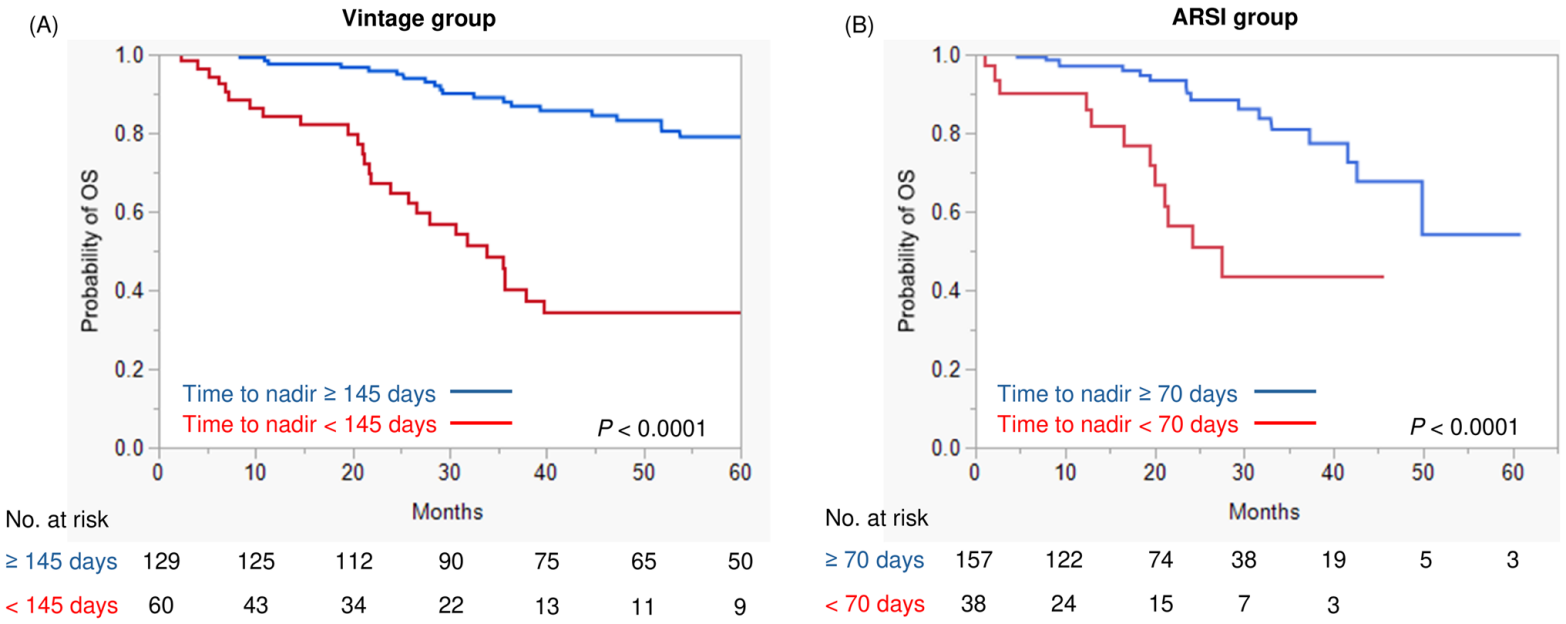
Survival analysis divided by optimal cut-off for time to PSA nadir. **A** Vintage treatment group. **B** ARSI treatment group

Kaplan–Meier analysis showed that patients who achieved a PSA nadir ≤ 1.5 ng/ml had prolonged OS compared to those who did not in the vintage group (*P* < 0.0001) (Fig. 3A). In the ARSI group, patients who achieved a PSA nadir ≤ 0.45 ng/ml had better OS than those who did not (*P* < 0.0001) (Fig. 3B). Regarding analysis of time to PSA nadir, men with time to nadir ≥ 145 days was associated with improved OS than time to nadir < 145 days in the vintage group (*P* < 0.0001) (Fig. 4A). Furthermore, a time to nadir of 70 days allowed significant stratification of prognosis in the ARSI group (*P* < 0.0001) (Fig. 4B). Notably, the prognosis of men who achieved PSA nadir ≤ 0.45 ng/ml in the ARSI group was similar to that of patients with PSA nadir ≤ 1.5 ng/ml in the vintage group (3-year survival was 85.1% and 87.7%, respectively: *P* = 0.8582) (Fig. S1). Furthermore, the prognosis of patients who did not have favorable response was similar between the ARSI and vintage groups (*P* = 0.8835) (Fig. S1). In addition, we validated the prognostic impact of the same PSA nadir level and time to nadir in both treatment groups (Fig. S2−5). No significant differences in OS were observed between the ARSI and vintage treatment when the same cut-off was used.

### PSA kinetics as a prognostic factor for OS

To examine the prognostic impact of PSA kinetics for OS, we performed Cox proportional hazard modeling using other clinical factors. In the ARSI treatment group, elevated LDH (> 194U/L), PSA nadir (> 0.45 ng/ml), and time to PSA nadir (> 70 days) were identified as predictive factors for OS in univariate analysis (Table 3). After multivariate analysis, PSA nadir (> 0.45 ng/ml) was the only independent predictive factor for OS (hazard ratio (HR) = 4.66, 95% confidence interval (CI): 1.96–12.35, *P* = 0.0004) (Table 3).

**Table 3:** Uni- and multivariate cox proportional hazard models for OS in ARSI group

	Univariate			Multivariate		
	HR	95% CI	*P* value	HR	95% CI	*P* value
Age (> 74)	1.19	0.57–2.42	0.6448	-	-	-
initial PSA (> 209.5)	1.2	0.59–2.52	0.6158	-	-	-
ISUP GG5	2.06	0.96–4.92	0.0638	-	-	-
clinical T stage 4	1.73	0.83–3.62	0.1439	-	-	-
Liver metastasis	1.42	0.08–6.70	0.742	-	-	-
EOD ≥ 2	1.83	0.88–4.05	0.106	-	-	-
Hb (< 13.3)	1.01	0.47–2.13	0.9883	-	-	-
LDH (> 194)	2.78	0.15–0.78	0.0091	0.49	0.20–1.12	0.0911
ALP (> 261)	1.01	0.48–2.19	0.9799	-	-	-
Alb (< 4)	1.02	0.49–2.14	0.9482	-	-	-
PSA nadir > 0.45 ng/ml	6.03	2.77–14.49	< 0.0001	4.66	1.96–12.35	0.0004
time to PSA nadir > 70 days	4.25	1.96–8.95	0.0004	2.1	0.88–4.83	0.091
high volume	1.25	0.58–2.98	0.5811	-	-	-
high risk	1.01	0.46–2.56	0.9735	-	-	-

*OS* overall survival, *HR* hazard ratio, *CI* confidence interval, *PSA* prostate-specific antigen, *ISUP GG* International Society of Urological Pathology grade group, *Hb* hemoglobin, *LDH* lactate dehydrogenase, *ALP* alkaline phosphatase, *Alb* albumin

In the vintage treatment group, clinical T stage 4 (HR = 1.97, 95%CI: 1.12–3.45, *P* = 0.02), EOD score ≥ 2 (HR = 1.83, 95%CI: 1.01–3.31, *P* = 0.046), PSA nadir (> 1.5 ng/ml) (HR = 2.39, 95%CI: 1.26–4.53, *P* = 0.0075), and time to PSA nadir (> 145 days) (HR = 3.88, 95%CI: 2.1–7.16, *P* < 0.0001) were independent predictive factors for OS in multivariate analysis (Table 4).

**Table 4:** Uni- and multivariate cox proportional hazard models for OS in Vintage group

	Univariate			Multivariate		
	HR	95% CI	*P* value	HR	95% CI	*P* value
Age (> 75)	1.49	0.89–2.48	0.1205	-	-	-
initial PSA (> 169.98)	1.35	0.82–2.26	0.2419	-	-	-
ISUP GG5	2.98	1.58–6.22	0.0004	1.28	0.62–2.64	0.493
clinical T stage 4	1.71	1.01–2.86	0.0445	1.97	1.12–3.45	0.02
Liver metastasis	3.92	0.63–13.00	0.1207	-	-	-
EOD ≥ 2	2.77	1.63–4.78	0.0002	1.83	1.01–3.31	0.046
Hb (< 13.5)	0.88	0.53–1.47	0.6225	-	-	-
LDH (> 192)	1.37	0.82–2.31	0.2366	-	-	-
ALP (> 451.7)	1.35	0.80–2.33	0.2589	-	-	-
Alb (< 4.1)	0.93	0.54–1.63	0.8087	-	-	-
PSA nadir > 1.5 ng/ml	5.01	2.94–8.59	< 0.0001	2.39	1.26–4.53	0.0075
time to PSA nadir > 145 days	5.05	2.96–8.69	< 0.0001	3.88	2.10–7.16	< 0.0001
high volume	1.62	0.96–2.83	0.0704	-	-	-
high risk	1.59	0.92–2.87	0.0977	-	-	-

*OS* overall survival, *HR* hazard ratio, *CI* confidence interval, *PSA* prostate-specific antigen, *ISUP GG* International Society of Urological Pathology grade group, *Hb* hemoglobin, *LDH* lactate dehydrogenase, *ALP* alkaline phosphatase, *Alb* albumin

### Characteristics of patients with poor PSA response associated with worse survival

**Fig. 5:**
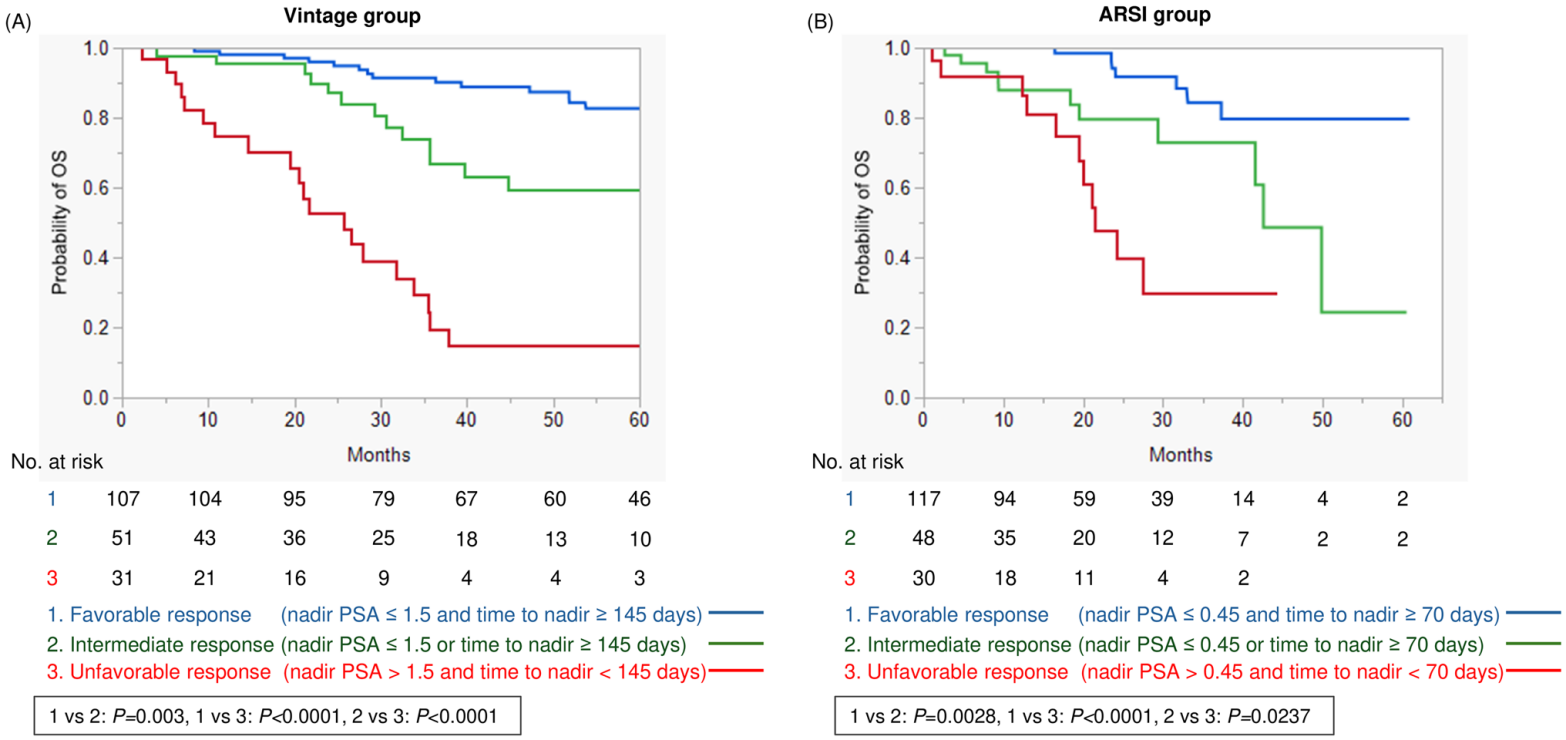
Survival analysis by risk classification using PSA nadir and time to PSA nadir. **A** Vintage treatment group. **B** ARSI treatment group

In addition, a novel risk classification was established using PSA nadir level and time to nadir period in each treatment group. We divided patients into Favorable (presence of both favorable factors), Intermediate (presence of only one favorable factor), and Unfavorable (presence of no favorable factors) groups. Kaplan–Meier analysis revealed that our risk classification allowed distinct stratification of survival outcomes for patients receiving vintage treatment (Fig. 5A). The Favorable group showed a significantly decreased risk of death as compared to the Intermediate (*P* = 0.003) and Unfavorable (*P* < 0.0001) response groups. Likewise in the ARSI treatment group, these risk classifications were able to stratify patient prognosis (Favorable vs Intermediate: *P* = 0.0028; Intermediate vs Unfavorable: *P* = 0.0237) (Fig. 5B).

To identify the clinical factors correlated with poor PSA response and shorter survival, we further examined the characteristics of patients classified by our risk groups in both treatment groups. In the ARSI treatment group, men with Intermediate or Unfavorable responses showed higher rates of EOD score ≥ 2 as compared to men with Favorable response (*P* = 0.0226) (Table 5). The Favorable group had a higher frequency of M1c stage (*P* = 0.0044) due to lung metastasis (*P* = 0.0015), which may have resulted in favorable outcomes (Table 5). No significant differences in age, iPSA level, ISUP GG, baseline values of peripheral blood markers, disease volume/risk, or types of ARSI drugs were found between Favorable and Intermediate/Unfavorable groups (Table 5). On the other hand, ISUP GG and frequency of clinical T stage4 tended to be higher in the Intermediate/Unfavorable groups than Favorable group (*P* = 0.079 and *P* = 0.0778, respectively).

**Table 5:** Characteristics of patients regarding PSA response after ARSI treatment

	Groups		*P* value*
	Favorable (*n* = 118)	Intermediate + Unfavorable (*n* = 78)	
Median age (range), years	74.5 (58–88)	74 (52–91)	0.7264
Median initial PSA (range), ng/ml	186.744 (5.18–17,300)	286 (4.9–9365)	0.2631
ISUP GG, *n* (%) ≤ 3	4 (3.4)	3 (3.8)	0.079*
4	42 (35.6)	18 (23.1)	
5	72 (61)	57 (73.1)	
T stage, *n* (%) ≤ 3b	84 (71.2)	46 (58.9)	0.0778
≥ 4	34 (28.8)	32 (41.1)	
N stage, *n* (%)			0.5946
positive M stage, *n* (%) 1a/1b/1c	56 (47.5) 10 (8.5)/ 77 (65.3)/ 31(26.3)	34 (43.6) 5 (6.4)/ 65 (83.3)/ 8 (10.3)	0.0044^+^
EOD score ≥ 2	47 (39.8)	44 (56.4)	0.0226
Location of visceral metastasis, *n* (%)			
Liver	2 (1.7)	3 (3.8)	0.3564
Lung	29 (24.6)	6 (7.7)	0.0015
Both	1 (0.8)	3 (3.8)	0.1485
Baseline peripheral blood markers, median			
Hb (range), g/dL	13.2 (5.5–17.3)	13.5 (7.1–17.7)	0.464
LDH (range), U/L	195 (133–1715)	188.5 (128–2189)	0.9781
ALP (range), U/L	252.04 (55–9813)	261 (55–3714.7)	0.5346
Alb (range), g/dL	4.05 (2.6–4.9)	4 (2.5–4.9)	0.3219
High volume, *n* (%)	73 (61.9)	49 (62.8)	0.8925
High risk, *n* (%)	81 (68.6)	53 (67.9)	0.9184
initial treatment, *n* (%)			
Apalutamide	29 (24.6)	23 (29.5)	0.4475
Enzalutamide	21 (17.8)	13 (16.7)	0.8377
Abiraterone acetate	68 (57.6)	42 (53.8)	0.6018

*ARSI* androgen receptor signaling inhibitor, *PSA* prostate-specific antigen, *ISUP GG* International Society of Urological Pathology grade group, *EOD* extent of disease, *Hb* hemoglobin, *LDH* lactate dehydrogenase, *ALP* alkaline phosphatase, *Alb* albumin, * frequency of GG5, ^*+*^ frequency of M1c

In the vintage treatment group, ISUP GG5 (*P* < 0.0001), EOD score ≥ 2 (*P* = 0.0031), high-volume disease (*P* = 0.038), and high-risk disease (*P* = 0.0344) were more likely to be observed in the Intermediate/Unfavorable groups in comparison with the Favorable group (Table 6). No significant differences in age, iPSA, tumor stage, or baseline values of peripheral blood markers were seen (Table 6).

**Table 6:** Characteristics of patients regarding PSA response after Vintage treatment

	Groups		*P* value*
	Favorable (*n* = 107)	Intermediate + Unfavorable (*n* = 82)	
Median age (range), years	75 (51–89)	75.5 (54–93)	0.1528
Median initial PSA (range), ng/ml	159.8 (2.65–13,050)	194.0295 (1.15–8891.75)	0.2248
ISUP GG, *n* (%) ≤ 3	5 (4.7)	0 (0)	< 0.0001*
4	44 (41.1)	13 (15.9)	
5	58 (54.2)	69 (84.1)	
T stage, *n* (%) ≤ 3b	67 (62.6)	53 (64.6)	0.7751
≥ 4	40 (37.4)	29 (35.4)	
N stage, *n* (%)			0.2671
positive M stage, *n* (%) 1a/1b/1c	62 (57.9) 8 (7.5)/80 (74.8)/19 (17.8)	54 (65.9) 5 (6.1)/65 (79.3)/12 (14.6)	0.564^+^
EOD score ≥ 2	37 (34.6)	46 (56.1)	0.0031
Location of visceral metastasis, *n* (%)			
Liver	21 (19.6)	12 (14.6)	0.3672
Lung	1 (0.9)	4 (3.9)	0.0892
Both	0 (0)	2 (2.4)	0.0665
Baseline peripheral blood markers, median			
Hb (range), g/dL	13.5 (5.5–18.8)	13.45 (7.4–16.7)	0.9784
LDH (range), U/L	185.5 (125–1715)	208 (130–545)	0.7677
ALP (range), U/L	392 (86–14,290.88)	474.14 (86–4989.88)	0.3253
Alb (range), g/dL	4.1 (2.6–4.9)	4.1 (2.1–5.1)	0.6783
High volume, *n* (%)	60 (56.1)	58 (70.7)	0.038
High risk, *n* (%)	64 (59.8)	61 (74.4)	0.0344

*ARSI* androgen receptor signaling inhibitor, *PSA* prostate-specific antigen, *ISUP GG* International Society of Urological Pathology grade group, *EOD* extent of disease, *Hb* hemoglobin, *LDH* lactate dehydrogenase, *ALP* alkaline phosphatase, *Alb* albumin, * frequency of GG5, ^*+*^ frequency of M1c

## Discussion

Our observational study included 213 patients in the ARSI group and 213 patients in the vintage group with background characteristics unified by the PSM to examine PSA kinetics and oncological outcomes in mHSPC disease. These analyses found no significant differences in PSA reduction at 3 months or PSA nadir level between the ARSI and vintage treatment groups, although the ARSI group tended to show faster and deeper response. Regarding time to PSA nadir, vintage treatment had a slower PSA decline as compared to ARSI treatment, particularly in progression-free cases. In addition, we examined the optimal cut-off PSA nadir and time to PSA nadir to predict OS in each treatment group, revealing distinct thresholds using survival tree analysis. Different from receiver operating characteristic (ROC) curve, this analysis can consider both prognosis and time to event. ARSI treatment requires earlier and deeper PSA dynamics than vintage treatment to assure longer OS. Multivariate analysis highlighted the importance of our calculated PSA kinetics as a prognostic factor. In addition, we established a classification based on PSA nadir level and time to nadir and stratified patient prognosis. After analyzing the background characteristics of patients, larger number of bone metastases was associated with poor PSA response in the ARSI group. In the vintage group, higher GG, larger number of bone metastases, and high-volume/high-risk were correlated with worse PSA response, indicating that these populations are likely to relapse earlier and require more intensive first-line treatment. Thus, PSA dynamics can be a useful surrogate marker based on our findings and offer early suggestions for changes in treatment sequence. These findings will facilitate the development of better PSA observation and detection of unfavorable PSA response in patients in the early stages of treatment.

PSA dynamics have been considered as a prognostic marker based on the idea that initial responsiveness to ADT is related to treatment effect even after CRPC development and determines OS [19, 20]. A lower PSA nadir value (≤ 0.64 ng/ml) and longer time to nadir (≥ 7 months) for initial ADT as calculated from the ROC curve were associated with favorable prognosis after the development of CRPC [13]. Achievement of an ultra-low PSA nadir of ≤ 0.02 ng/ml after apalutamide treatment for patients with mHSPC has been correlated with a decreased risk of death [21]. Although many studies have elucidated favorable PSA reduction with ARSIs and an association with prognosis, few studies have examined the extent to which PSA reduction is actually associated with a better prognosis. Our study revealed comparable prognosis between men with a PSA nadir of ≤ 1.5 ng/ml under vintage treatment and men with a PSA nadir of ≤ 0.45 ng/ml under ARSI treatment, indicating that ARSI treatment requires deeper PSA response to ensure better subsequent survival. In addition, the optimal cut-off for time to PSA nadir in the ARSI treatment group was shorter (70 days) than that in the vintage treatment group (145 days). These findings suggest that earlier and more stringent thresholds are needed when patients received ARSI treatment in comparison with vintage treatment.

Enzalutamide (MDV3100) was developed as a second-generation antiandrogen agent and binds to the androgen receptor with a higher relative affinity than bicalutamide, reducing nuclear translocation efficiency and inhibiting both DNA binding to androgen response elements and recruitment of coactivators [22, 23]. Apalutamide (ARN-509) has a similar structure to enzalutamide and achieved comparable efficiency in xenograft models of CRPC [24]. Real-world data showed that these ARSIs offered more potent effects for PSA reduction and stabilization than bicalutamide in patients with mHSPC [25].

A randomized clinical trial investigated the efficiency of enzalutamide versus bicalutamide in combination with ADT in patients with mHSPC and found an improved 7-month PSA response rate in the enzalutamide group among Black patients (93% vs 42%, *P* = 0.009), although no significant benefit was seen in non-Black patients (94% vs 86%, not significant) [26]. In addition, a recent study identified that mHSPC patients were at low risk for disease progression as defined by PSA nadir and PSA change after bicalutamide treatment in addition to ADT, indicating that bicalutamide administration with close monitoring of PSA can eliminate the need for ARSI treatment in some populations [27]. Racial differences may, thus, exist in PSA-suppressing effects, and some patient populations can show similar effects on bicalutamide as on ARSI, supporting the present findings.

To the best of our knowledge, this study is the first to report on differences in PSA dynamics between ARSI and vintage (bicalutamide) treatment combining with ADT in men with mHSPC. Further, we identified the optimal cut-off PSA nadir level and time to nadir in both of these treatment groups, which will provide useful information for PSA monitoring in clinical practice.

The results of this study should be interpreted while keeping several limitations in mind. First, our analysis was performed retrospectively. Second, the number of patients was relatively small, and this study was conducted in a specific population in Japan. Lastly, the observation periods in this study were relatively short. Larger, prospective, cross-racial studies with long-term follow-up are, thus, needed to validate the present findings.

## Conclusion

The present study characterized the PSA dynamics of mHSPC patients treated with ARSIs or vintage agent (bicalutamide) in combination with ADT. Furthermore, we showed that the optimal cut-off values of PSA nadir and time to nadir for predicting improved survival differed between treatments. These findings suggest that earlier and deeper PSA responses are required to predict longer survival in ARSI treatment compared to bicalutamide. This study provides novel insights into understanding of PSA monitoring and early detection of patient populations at risk of unfavorable outcomes.

## Supplementary Information

Below is the link to the electronic supplementary material.

Supplementary file1—Figure S1. Survival analysis classified by optimal cut-off PSA nadir level in both treatment groups (TIFF 1925 KB)

Supplementary file2—Figure S2. Survival analysis classified by same PSA nadir level (0.45ng/ml) in both treatment groups (TIFF 1769 KB)

Supplementary file3—Figure S3. Survival analysis classified by same PSA nadir level (1.5ng/ml) in both treatment groups (TIFF 1726 KB)

Supplementary file4—Figure S4. Survival analysis classified by same time to PSA nadir (70days) in both treatment groups (TIFF 1810 KB)

Supplementary file5—Figure S5. Survival analysis classified by same time to PSA nadir (145days) in both treatment groups (TIFF 1815 KB)

Supplementary file6 (DOCX 14 KB)

Supplementary file7 (DOCX 11 KB)

Supplementary file8 (DOCX 11 KB)

## Data Availability

All data generated or analyzed during this study, which support the findings of this study, are included within this article. Researchers may access analyses not present in the manuscript from the corresponding author upon reasonable request.
