# Supplementary figures and images for: Characterization of PSA dynamics and oncological outcomes in patients with metastatic hormone-sensitive prostate cancer treated with androgen receptor signaling inhibitors
Source: Int J Clin Oncol. 2024 Dec 10;30(3):539–50. doi: 10.1007/s10147-024-02676-z (PMC11842405; doi:10.1007/s10147-024-02676-z)

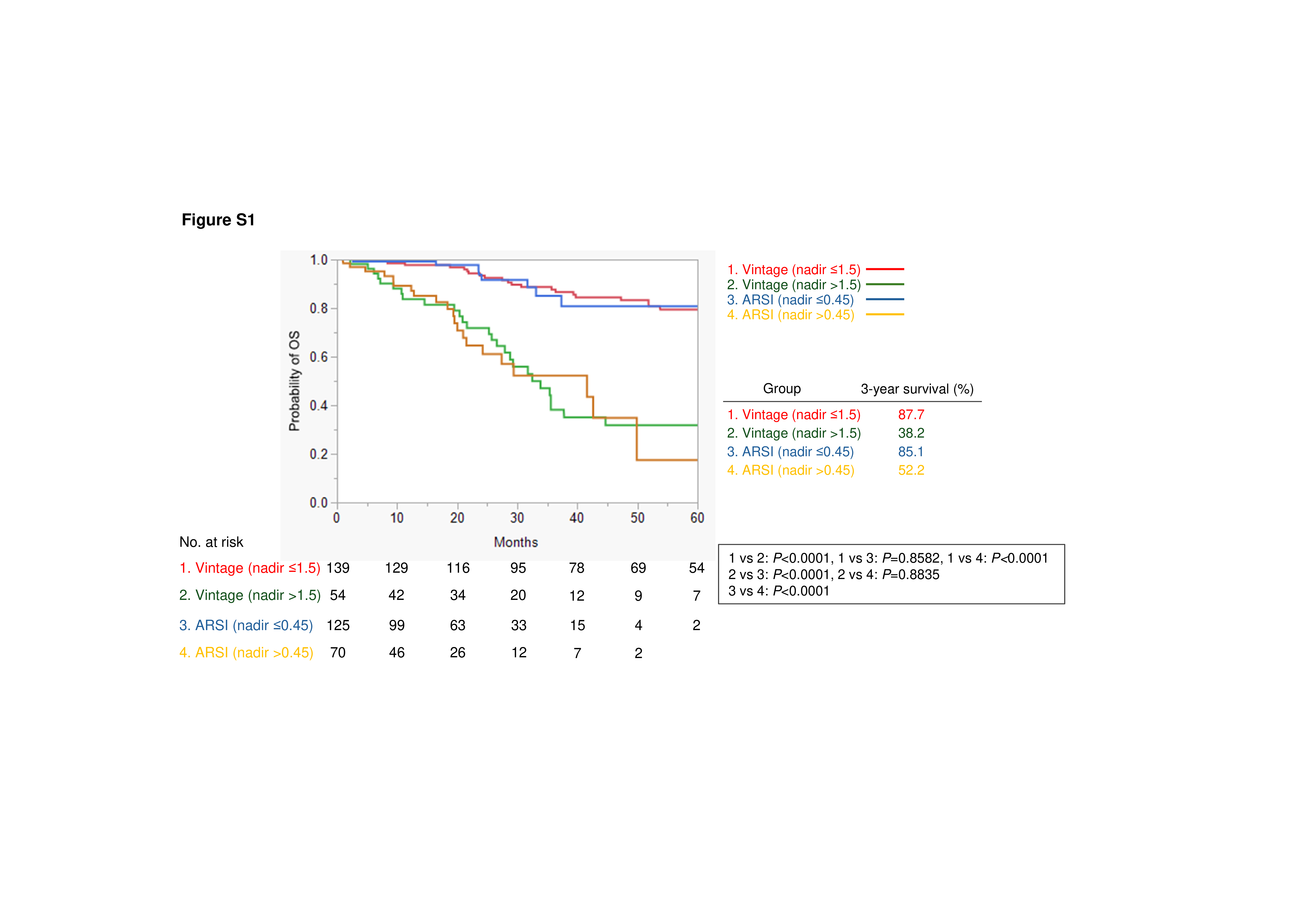

Supplement: Supplementary file 1 — Supplementary file1—Figure S1. Survival analysis classified by optimal cut-off PSA nadir level in both treatment groups (TIFF 1925 KB) [file 10147_2024_2676_MOESM1_ESM.tiff]

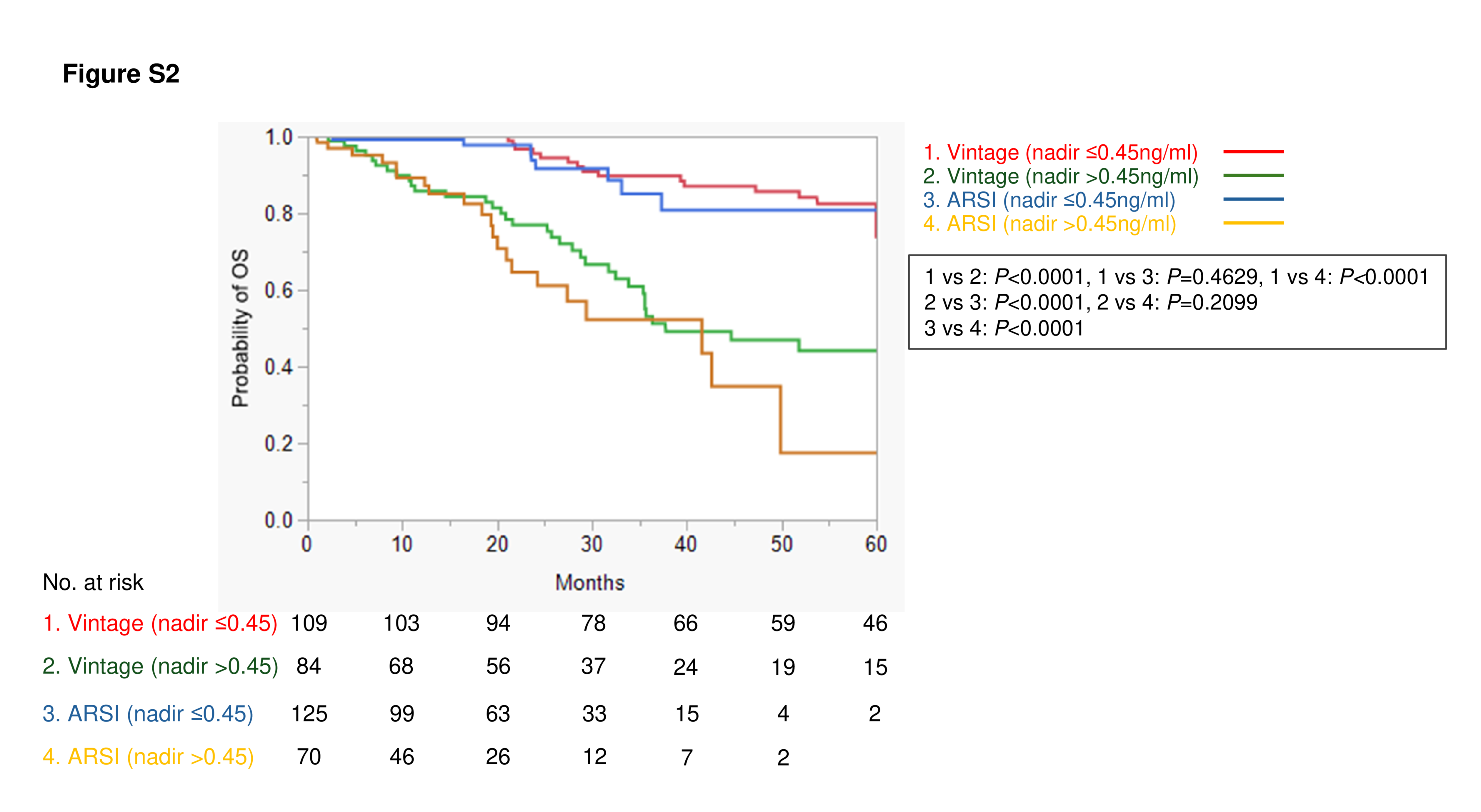

Supplement: Supplementary file 2 — Supplementary file2—Figure S2. Survival analysis classified by same PSA nadir level (0.45ng/ml) in both treatment groups (TIFF 1769 KB) [file 10147_2024_2676_MOESM2_ESM.tiff]

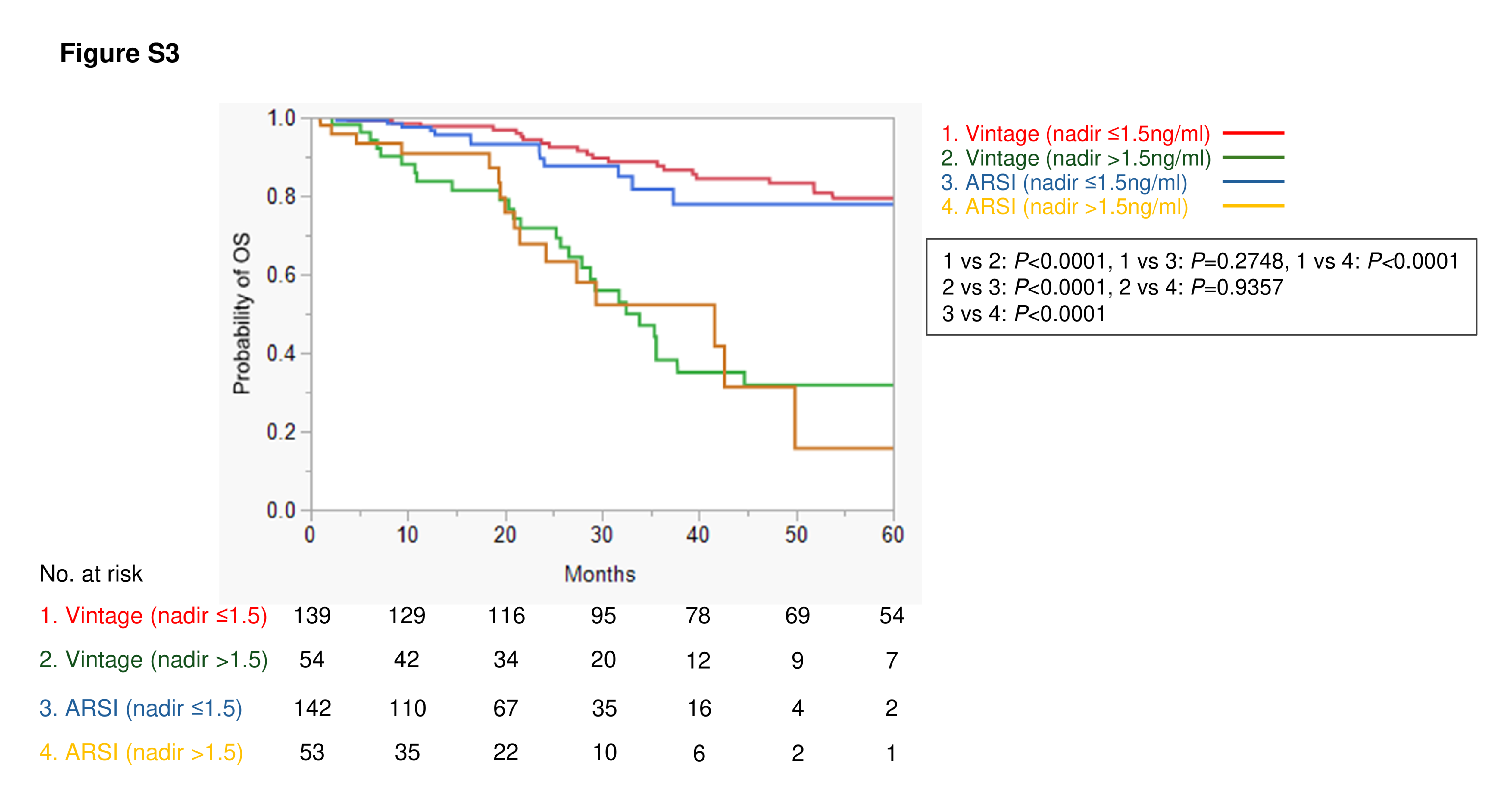

Supplement: Supplementary file 3 — Supplementary file3—Figure S3. Survival analysis classified by same PSA nadir level (1.5ng/ml) in both treatment groups (TIFF 1726 KB) [file 10147_2024_2676_MOESM3_ESM.tiff]

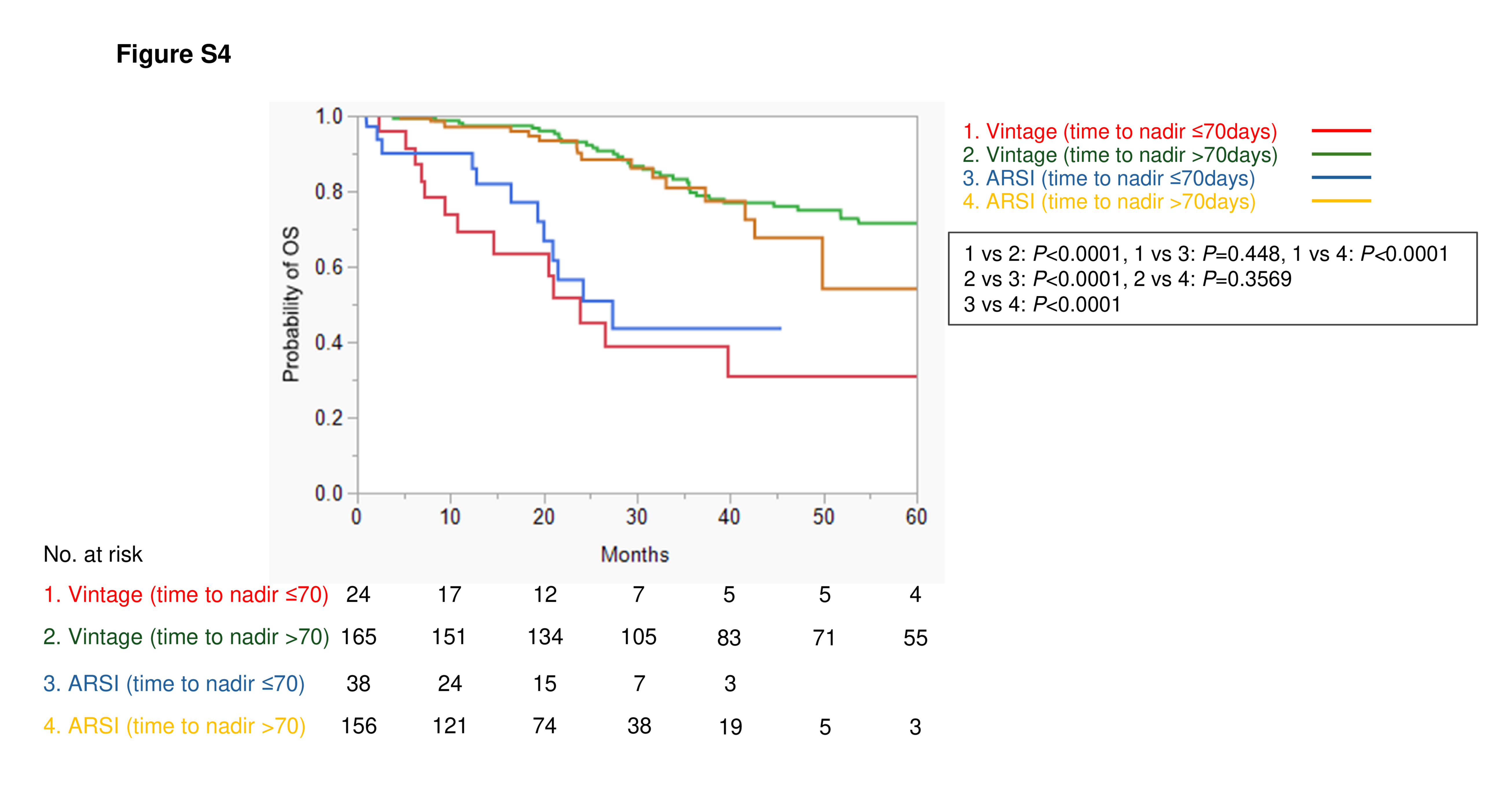

Supplement: Supplementary file 4 — Supplementary file4—Figure S4. Survival analysis classified by same time to PSA nadir (70days) in both treatment groups (TIFF 1810 KB) [file 10147_2024_2676_MOESM4_ESM.tiff]

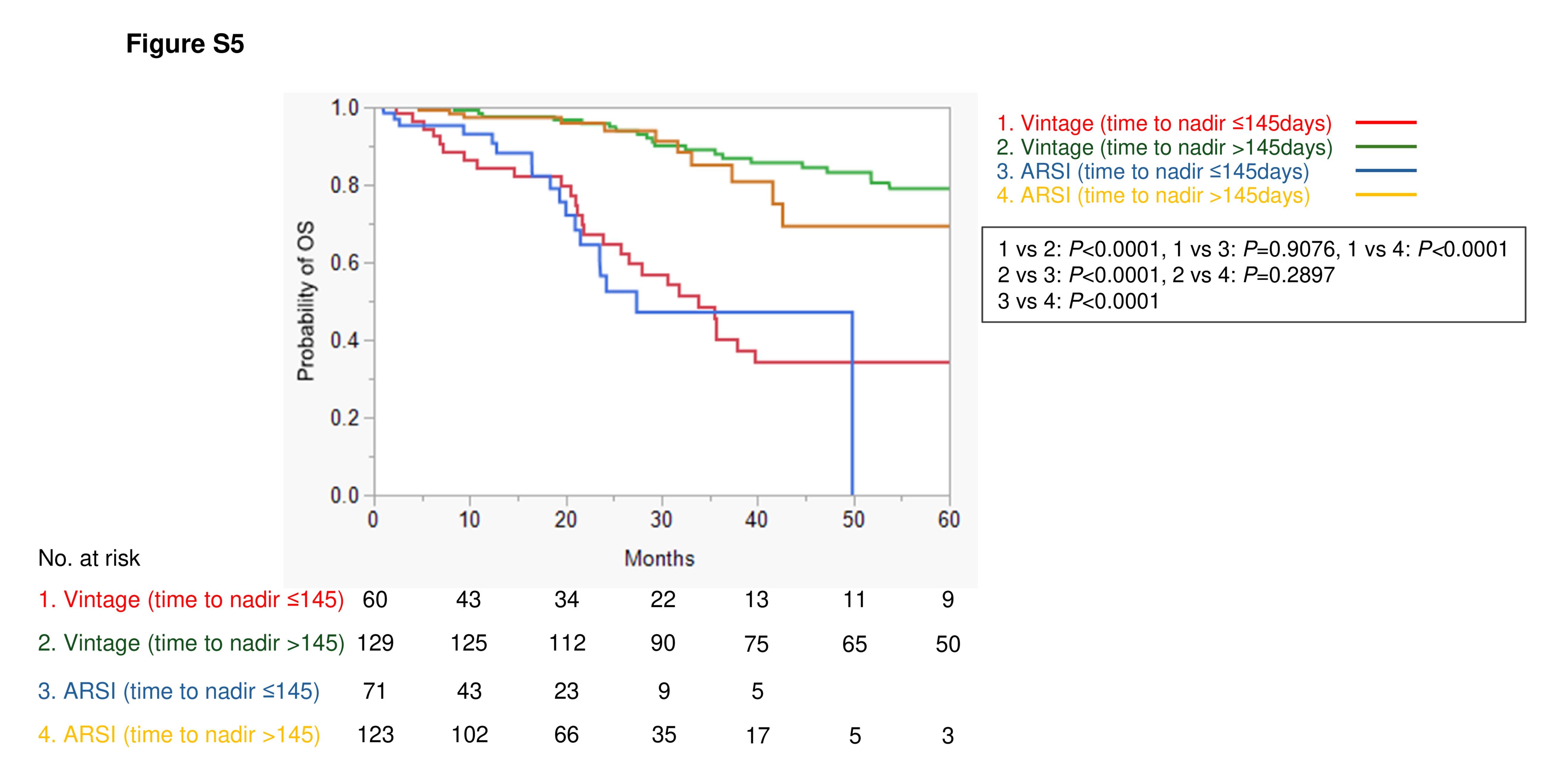

Supplement: Supplementary file 5 — Supplementary file5—Figure S5. Survival analysis classified by same time to PSA nadir (145days) in both treatment groups (TIFF 1815 KB) [file 10147_2024_2676_MOESM5_ESM.tiff]
