# Supplementary material for: Characterization of PSA dynamics and oncological outcomes in patients with metastatic hormone-sensitive prostate cancer treated with androgen receptor signaling inhibitors
Source: Int J Clin Oncol. 2024 Dec 10;30(3):539–50. doi: 10.1007/s10147-024-02676-z (PMC11842405; doi:10.1007/s10147-024-02676-z)
Supplement: Supplementary file 6 — Supplementary file6 (DOCX 14 KB) [file 10147_2024_2676_MOESM6_ESM.docx]

Table S1. Patients' characteristics of original cohort.

Treatment groups

*P* value

|  | ARSI (n = 304) | Vintage (n = 295) |  |
| --- | --- | --- | --- |
| Median age (range), years | 74 (45-91) | 75 (51-93) | 0.0011 |
| Median initial PSA (range), ng/ml | 220 (4.145-17300) | 173.77 (1.15-19908) | 0.9157 |
| ISUP GG, n (%)  ≤ 3 | 17 (5.6) | 8 (2.7) | 0.837^*^ |
| 4 | 80 (26.3) | 69 (23.4) |  |
| 5 | 183 (60.2) | 151 (51.2) |  |
| T stage, n (%) |  |  | 0.2386 |
| ≤ 3b | 189 (62.2) | 197 (66.8) |  |
| ≥ 4 | 115 (37.8) | 98 (33.2) |  |
| N stage, n (%) |  |  | <0.0001 |
| positive | 245 (80.6) | 167 (56.6) |  |
| M stage, n (%) |  |  | 0.0001^+^ |
| 1a/1b/1c | 26 (8.6)/191 (62.8)/80 (26.3) | 16 (5.4)/219 (74.2)/41 (13.9) |  |
| EOD score |  |  | 0.2273 |
| ≥ 2 | 151 (49.7) | 132 (44.7) |  |
| Location of visceral metastasis, n (%) |  |  |  |
| Liver | 8 (2.6) | 6 (2) | 0.6277 |
| Lung | 70 (23) | 45 (15.3) | 0.0154 |
| Both | 6 (1.9) | 3 (1) | 0.3309 |
| Baseline peripheral blood markers, median |  |  |  |
| Hb (range), g/dL | 13.4 (5.5-19.7) | 13.4 (5.5-18.8) | 0.9571 |
| LDH (range), U/L | 195 (38-2189) | 193.5 (114-1715) | 0.4585 |
| ALP (range), U/L | 268 (52-10982.28) | 463.38 (70-14290.88) | 0.0083 |
| Alb (range), g/dL | 4 (2.5-14) | 4.1 (2.1-14) | 0.3797 |
| High volume, n (%) | 210 (69.1) | 167 (56.6) | 0.0016 |
| High risk, n (%) | 211 (69.4) | 166 (56.3) | 0.0009 |
| initial treatment, n (%) |  |  |  |
| Apalutamide | 89 (29.3) | 0 (0) | - |
| Enzalutamide | 61 (20.1) | 0 (0) | - |
| Abiraterone acetate | 154 (50.6) | 0 (0) | - |
| Bicalutamide | 0 (0) | 295 (100) | - |
| PSA progression, n (%) | 80 (26.3) | 160 (54.2) | - |
| Death, n (%) | 45 (14.8) | 103 (34.9) | - |

PSA: prostate-specific antigen; ISUP GG: International Society of Urological Pathology grade group; Hb: hemoglobin; LDH: lactate dehydrogenase; ALP: alkaline phosphatase; Alb: albumin; ARSI: androgen receptor signaling inhibitor; ^*^: frequency of GG5, ^+^: frequency of M1c
