# Supplementary material for: Characterization of PSA dynamics and oncological outcomes in patients with metastatic hormone-sensitive prostate cancer treated with androgen receptor signaling inhibitors
Source: Int J Clin Oncol. 2024 Dec 10;30(3):539–50. doi: 10.1007/s10147-024-02676-z (PMC11842405; doi:10.1007/s10147-024-02676-z)
Supplement: Supplementary file 7 — Supplementary file7 (DOCX 11 KB) [file 10147_2024_2676_MOESM7_ESM.docx]

Table S2. PSA dynamics in each treatment group in patients with iPSA > 200 ng/ml.

|  | Treatme | nt groups | *P* value* |
| --- | --- | --- | --- |
|  | ARSI | Vintage |  |
| PSA level at 3 months (ng/ml) |  |  |  |
| median (range) | 0.708 (0.01-1381) | 4.413 (0.007-1190) | 0.4074 |
| < 0.02 ng/ml | 2/87 (2.3%) | 3/85 (3.5%) | 0.63 |
| < 0.1 ng/ml | 12/87 (13.8%) | 6/85 (7.1%) | 0.1454 |
| < 0.2 ng/ml | 19/87 (21.8%) | 9/85 (10.6%) | 0.0436 |
| < 0.5 ng/ml | 39/87 (44.8%) | 16/85 (18.8%) | 0.0002 |
| < 1 ng/ml | 48/87 (55.2%) | 23/85 (27.1%) | 0.0002 |
| < 2 ng/ml | 53/87 (60.9%) | 33/85 (38.8%) | 0.0036 |
| ≥ 2 ng/ml | 34/87 (39.1%) | 52/85 (61.2%) | 0.0036 |
| PSA reduction at 3 months (%) |  |  |  |
| median (range) | 99.86 (-255-99.99) | 99.37 (-54-99.99) | 0.3705 |
| ≥ 50% | 84/87 (96.6%) | 83/85 (97.6%) | 0.6679 |
| ≥ 70% | 80/87 (91.9%) | 82/85 (96.5%) | 0.1994 |
| ≥ 90% | 75/87 (86.2%) | 79/85 (92.9%) | 0.1454 |
| ≥ 99%  nadir PSA level (ng/ml) | 58/87 (66.7%) | 48/85 (56.5%) | 0.1688 |
| median (range) | 0.205 (0-4343.35) | 0.9 (0-534.6) | 0.1412 |
| < 0.02 ng/ml | 24/101 (23.8%) | 12/90 (13.3%) | 0.0632 |
| < 0.1 ng/ml | 41/101 (40.6%) | 25/90 (27.8%) | 0.062 |
| < 0.2 ng/ml | 50/101 (49.5%) | 29/90 (32.2%) | 0.0151 |
| < 0.5 ng/ml | 58/101 (57.4%) | 36/90 (40%) | 0.0159 |
| < 1 ng/ml | 62/101 (61.4%) | 48/90 (53.3%) | 0.2609 |
| < 2 ng/ml | 67/101 (66.3%) | 58/90 (64.4%) | 0.7837 |
| ≥ 2 ng/ml  time to nadir (days), median (range) | 34/101 (33.7%) | 32/90 (35.6%) | 0.7837 |
| all | 203 (0-1035) | 203 (0-1472) | 0.0949 |
| progression cases | 199 (0-624) | 176 (0-646) | 0.4097 |
| progression-free cases | 231 (7-1035) | 353 (66-1472) | < 0.0001 |

PSA: prostate-specific antigen; ARSI: androgen receptor signaling inhibitor
