# Supplementary material for: Characterization of PSA dynamics and oncological outcomes in patients with metastatic hormone-sensitive prostate cancer treated with androgen receptor signaling inhibitors
Source: Int J Clin Oncol. 2024 Dec 10;30(3):539–50. doi: 10.1007/s10147-024-02676-z (PMC11842405; doi:10.1007/s10147-024-02676-z)
Supplement: Supplementary file 8 — Supplementary file8 (DOCX 11 KB) [file 10147_2024_2676_MOESM8_ESM.docx]

Table S3. PSA dynamics in each treatment group in patients with iPSA ≤ 200 ng/ml.

|  | Treatme | nt groups | *P* value* |
| --- | --- | --- | --- |
|  | ARSI | Vintage |  |
| PSA level at 3 months (ng/ml) |  |  |  |
| median (range) | 0.4365 (0.008-59) | 0.448 (0.007-652) | 0.2015 |
| < 0.02 ng/ml | 7/84 (8.3%) | 7/94 (7.4%) | 0.8265 |
| < 0.1 ng/ml | 26/84 (30.9%) | 29/94 (30.9%) | 0.9883 |
| < 0.2 ng/ml | 31/84 (36.9%) | 35/94 (37.2%) | 0.9638 |
| < 0.5 ng/ml | 45/84 (53.6%) | 50/94 (53.2%) | 0.9595 |
| < 1 ng/ml | 54/84 (64.3%) | 56/94 (59.6%) | 0.5181 |
| < 2 ng/ml | 69/84 (82.1%) | 65/94 (69.1%) | 0.0431 |
| ≥ 2 ng/ml | 15/84 (17.9%) | 29/94 (30.6%) | 0.0431 |
| PSA reduction at 3 months (%) |  |  |  |
| median (range) | 99.09 (28.14-99.9) | 99.05 (-332.93-99.99) | 0.1127 |
| ≥ 50% | 82/84 (97.6%) | 86/94 (91.5%) | 0.0657 |
| ≥ 70% | 80/84 (95.2%) | 83/94 (88.3%) | 0.0891 |
| ≥ 90% | 73/84 (86.9%) | 79/94 (84%) | 0.5885 |
| ≥ 99%  nadir PSA level (ng/ml) | 44/84 (52.3%) | 48/94 (51.1%) | 0.8606 |
| median (range) | 0.057 (0-96) | 0.085 (0-139.781) | 0.6355 |
| < 0.02 ng/ml | 39/95 (41.1%) | 38/103 (36.9%) | 0.5487 |
| < 0.1 ng/ml | 53/95 (55.8%) | 54/103 (52.4%) | 0.6352 |
| < 0.2 ng/ml | 59/95 (62.1%) | 61/103 (59.2%) | 0.6783 |
| < 0.5 ng/ml | 71/95 (74.7%) | 74/103 (71.8%) | 0.6459 |
| < 1 ng/ml | 76/95 (80%) | 80/103 (77.7%) | 0.6884 |
| < 2 ng/ml | 82/95 (86.3%) | 87/103 (84.5%) | 0.7128 |
| ≥ 2 ng/ml  time to nadir (days), median (range) | 13/95 (13.7%) | 16/103 (15.5%) | 0.7128 |
| all | 196 (28-1620) | 202 (0-2298) | 0.1224 |
| progression cases | 172.5 (35-378) | 168.5 (0-987) | 0.2663 |
| progression-free cases | 203 (28-1620) | 207 (0-2298) | 0.0777 |

PSA: prostate-specific antigen; ARSI: androgen receptor signaling inhibitor
